# Dried Blood Spots, an Affordable Tool to Collect, Ship, and Sequence gDNA from Patients with an X-Linked Agammaglobulinemia Phenotype Residing in a Developing Country

**DOI:** 10.3389/fimmu.2018.00289

**Published:** 2018-02-16

**Authors:** Gesmar R. S. Segundo, Anh T. V. Nguyen, Huyen T. Thuc, Le N. Q. Nguyen, Roger H. Kobayashi, Hai T. Le, Huong T. M. Le, Troy R. Torgerson, Hans D. Ochs

**Affiliations:** ^1^University of Washington and Seattle Children’s Research Institute, Seattle, WA, United States; ^2^Department of Pediatrics, Universidade Federal de Uberlandia, Uberlandia, Brazil; ^3^National Children’s Hospital, Hanoi, Vietnam; ^4^UCLA School of Medicine, Los Angeles, CA, United States

**Keywords:** X-linked agammaglobulinemia, genetic sequencing, primary immunodeficiencies, Btk, dried blood spots

## Abstract

**Background:**

New sequencing techniques have revolutionized the identification of the molecular basis of primary immunodeficiency disorders (PID) not only by establishing a gene-based diagnosis but also by facilitating defect-specific treatment strategies, improving quality of life and survival, and allowing factual genetic counseling. Because these techniques are generally not available for physicians and their patients residing in developing countries, collaboration with overseas laboratories has been explored as a possible, albeit cumbersome, strategy. To reduce the cost of time and temperature-sensitive shipping, we selected Guthrie cards, developed for newborn screening, to collect dried blood spots (DBS), as a source of DNA that can be shipped by regular mail at minimal cost.

**Method:**

Blood was collected and blotted onto the filter paper of Guthrie cards by completely filling three circles. We enrolled 20 male patients with presumptive X-linked agammaglobulinemia (XLA) cared for at the Vietnam National Children’s Hospital, their mothers, and several sisters for carrier analysis. DBS were stored at room temperature until ready to be shipped together, using an appropriately sized envelope, to a CLIA-certified laboratory in the US for sequencing. The protocol for Sanger sequencing was modified to account for the reduced quantity of gDNA extracted from DBS.

**Result:**

High-quality gDNA could be extracted from every specimen. Bruton tyrosine kinase (BTK) mutations were identified in 17 of 20 patients studied, confirming the diagnosis of XLA in 85% of the study cohort. Type and location of the mutations were similar to those reported in previous reviews. The mean age when XLA was suspected clinically was 4.6 years, similar to that reported by Western countries. Two of 15 mothers, each with an affected boy, had a normal BTK sequence, suggesting gonadal mosaicism.

**Conclusion:**

DBS collected on Guthrie cards can be shipped inexpensively by airmail across continents, providing sufficient high-quality gDNA for Sanger sequencing overseas. By using this method of collecting gDNA, we were able to confirm the diagnosis of XLA in 17 of 20 Vietnamese patients with the clinical diagnosis of agammaglobulinemia.

## Introduction

Recent technical advances in gene analysis have facilitated diagnosis and treatment of primary immunodeficiency diseases (PID), with more than 320 genes identified that cause PID when mutated (1). While those technologies are readily available in developed countries, most developing countries do not have the medical facilities to perform gene sequencing and protein analysis (2, 3). Many physicians caring for PID patients in these parts of the world enter into partnerships with researchers in the United States, Europe, and selected Asian countries to initiate genetic evaluation of their patients (2, 3). However, shipping blood or cryopreserved peripheral blood mononuclear cells between continents is hampered by bureaucratic road blocks, is expensive, and is sometimes unsuccessful.

**Figure 1:**
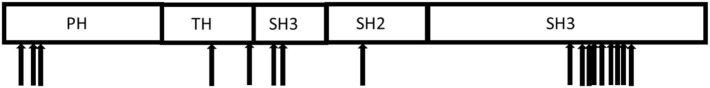
Distribution of the mutations in the Bruton tyrosine kinase gene identified in the 17 Vietnamese X-linked agammaglobulinemia patients.

X-linked agammaglobulinemia (XLA), a genetic disorder first described by Bruton in 1952 (4), is caused by mutations in the Bruton tyrosine kinase (*BTK*) gene (5). Patients with XLA have low or absence of circulating B cells and markedly reduced serum immunoglobulin and specific antibody levels, resulting in increased susceptibility to bacterial and selective viral infections. Affected boys present typically with respiratory tract infections, but may also develop colitis, arthritis, and neurologic findings (6–8). The *BTK* gene consists of 18 coding exons and directs the production of a 659 amino acid protein with 5 distinct structural domains (Figure 1) that plays a crucial role in B cell development and function (6, 9).

The clinical criteria sufficient for a probable diagnosis of XLA include a male with less than 2% CD19^+^ B cells in whom other causes of hypogammaglobulinemia have been excluded, who has at least one of the following: onset of recurrent bacterial infections in the first 5 years of life, serum IgG, IgM and IgA that are more than 2 SD below normal for age, and poor responses to vaccines. The “definitive” diagnosis of XLA requires gene sequencing confirming a mutation in BTK and/or absence of BTK protein expression in monocytes or platelets (10). Because analysis of protein expression and sequencing of the *BTK* gene are not commonly available in developing countries, a definitive diagnosis of XLA cannot be established.

To provide an inexpensive and effective method of performing genetic analyses in patients from developing countries with clinical and laboratorial findings compatible with a diagnosis of XLA, we tested the procedure established more than 50 years ago to collect and ship blood samples from hospitals to State laboratories for newborn screening. Originally designed for early detection of metabolic disorders such as phenylketonuria (11), dried blood spots (DBS) collected on filter paper (Guthrie cards) have been used in recent years as a source of gDNA to screen for severe combined immune deficiency, based on the presence or absence of T cell receptor excision circles, representing recent thymic emigrants (12, 13). We used Standard Guthrie cards to collect DBS, which can be shipped in a regular envelop by air mail (20 cards/envelop) from Vietnam to a CLIA-approved laboratory in Seattle, WA, USA, where the DNA was extracted and sequenced for mutations in the *BTK* gene. The approach has been validated by the demonstration that gDNA, stored for years in DBS, is stable and suitable for sequence analysis (14).

## Materials and Methods

### Ethics Statement

The DBS analysis for BTK mutations was approved by the institutional review boards at Seattle Children’s Hospital and Vietnam National Children’s Hospital. Written informed consent for genetic testing was obtained from the parents of Vietnamese patients by local physicians.

### Patients

Twenty patients with a clinical diagnosis of XLA, cared for by physicians at the Vietnam National Children’s Hospital, Hanoi, were enrolled in the study from May to July 2017, and DBS were collected on Guthrie cards. A possible clinical diagnosis of XLA was based on the increased susceptibility to bacterial infections at an early age, low serum immunoglobulin levels, and marked decrease or absence of peripheral blood B lymphocytes (10). Mothers and sisters of these patients were invited to provide DBS for investigation of carrier status.

### Samples

Vietnamese physicians caring for the patients collected venous blood or blood from a finger stick and completely filled three circles on the paper filter cards (ID Biological, Greenville, SC, USA) used by the Washington State Department of Health for newborn screening. The samples were stored at room temperature until DBS from all 20 patients were collected and were shipped in a single standard envelope by regular air mail to the Immunology Diagnostic Laboratory at Seattle Children’s Research Institute. Upon arrival, gDNA was extracted from one of the three blood spots collected from each patient using the Quiagen DNA blood Mini Kit (Quiagen, Germantown, USA). The average yield was 3–20 ng/µl (total 150 µl). The unused DBS were stored at room temperature for future use.

### BTK Gene Analysis

The targeted Sanger sequencing protocol for regular DNA analysis was modified, as the final concentration of gDNA extracted from DBS (5–25 ng/μl) was lower than that obtained from peripheral blood. We optimized PCR amplification using only 2 μl of gDNA per exon or 5 µl gDNA for the longest exons or multiple exons based on the (modified) primers reported previously (15). Briefly, samples were amplified in 10 µl reactions containing 1 µl each of forward and reverse primers (5 mM each), 1 µl of dNTP mix, 1 µl of 10× buffer, 0.5 µl of MgSO_4_ (50 mM), and 0.1 µl of undiluted Taq DNA Polymerase. All exons were amplified under the same PCR conditions: denaturation at 94°C during 2 min and then 38 cycles at 94°C for 30 s, 55°C for 30 s, and 68°C for 1.5 min, with final extension at 68°C for 5 min. Amplicons were sequenced according to the standard Big Dye protocol, analyzed, and aligned using the Mutation Surveyor and BioEdit software to detect mutations in the coding sequence and exon/intron junctions of *BTK*. The nomenclature for mutations in BTK is according to guidelines by the Human Genome Variation Society as determined by Mutalyzer 2.0.26-Name checker (NM_000061.2).

## Results

Table 1 presents clinical findings and laboratorial data of the 20 patients and lists *BTK* mutations identified in 17 participants. The mean age at the onset of symptoms was 9.52 months (range, 1–27 months), and the mean age at diagnosis was 54.70 months (range, 6–153 months). There was no difference between those patients with BTK mutations and those patients without. The mean age when the diagnosis was confirmed by BTK genetic analysis was 84.50 months (range, 21–197 months). The diagnosis of XLA based on the clinical characteristics was suspected at the age of 6 months in an asymptomatic boy (p06) who had an older diseased brother with an XLA phenotype. All patients started to receive IVIG as soon as the diagnosis of XLA was suspected clinically.

**Table 1:** Clinical findings, laboratory results, and *BTK* mutations in 20 Vietnamese patients with agammaglobulinemia.

Patient number	Birth date	Onset of symptoms, age (months)	Age at diagnosis (months)	Age when genetic analysis was done (months)	IgA (g/l)	IgM (g/l)	IgG (g/l)	CD19/mm^3^	CD19 (%)	*BTK* mutation	Mutation reported previously
p01	20/09/2012	4	14	58	0.03	0.07	3.17	5	0	c.1735G>C; p.D579H	No
p02	11/10/2011	6	22	69	0.01	0.09	0.01	1	0	c.1908+2delTAAGTGCTT (splicing defect)	No
p03	21/04/2010	4	75	87	0.01	0.27	0.16	7	0	c.752G>A; p.W251*	YES
p04	12/04/2011	9	42	75	0.05	0.08	0.1	78	1	c.117_119del; p.Y40del	No
P05	25/06/2011	5	23	73	0.01	0.14	0.07	8	0	c.521-1G>A (splicing defect)	No
P06	07/01/2013	^a^	6	54	0.13	0.13	0.22	48	1	c.1744del; p.A582Lfs*5	Yes
P07	08/10/2015	6	11	21	0	0.05	0.01	0	0	c.763C>T; p.R255*	Yes
P08	04/07/2011	27	58	72	0.01	0.17	0.06	0	0	c.1713_1714 del; p.Y571*	No
P09	29/09/2011	9	59	70	0.01	0.01	0.16	0	0	c.1768A>T; p.I590F	Yes
P10	10/02/2001	1	153	197	0.01	0.01	0.06	0	0	c.1782del; p.K595Rfs*54	No
p11	14/01/2003	24	113	174	0.35	0.36	1.58	2	0.1	c.37C>T; p.R13*	Yes
p12	23/10/2008	14	61	105	0.02	0.12	0.02	1	0	c.1610del; p.V537Dfs*19	No
p13	06/06/2008	9	63	109	0.03	0.16	1.2	1	0	c.953C>T; p.S318F	Yes
p14	27/09/2010	6	62	82	0.01	0.01	1.7	15	0.4	c.1657del; p.S553Afs*3	No
p15	17/11/2010	4	72	80	0.03	0.19	0.12	52	1	c.124T>G; p.Y42D	No
p16	06/01/2013	20	51	54	0.01	0.15	0.88	52	1	c.627_628insA; p.P210Tfs*6	No
p17	04/03/2012	8	59	64	0.02	0.21	0.06	20	1	c.1651T>A; p.Y551N	No
p18	07/07/2014	4	25	36	0.02	0.39	0.2	0	0	No mutation^b^	–
p19	22/06/2012	12	50	61	0.18	0.23	1.28	1	0	No mutation^b^	–
p20	10/02/2005	9	79	149	0.01	0.82	0.62	0	0	No mutation^b^	–

*^a^Patient diagnosed before he developed symptoms*.

*^b^In exons and exon/intron junctions*.

**Figure 2:**
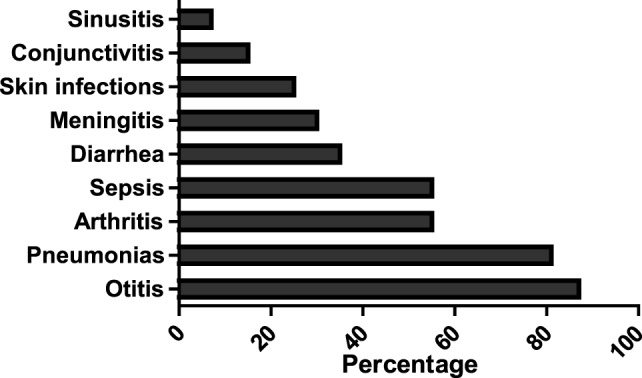
Frequency of infections in a cohort of 20 Vietnamese patients with agammaglobulinemia.

The most prevalent infections observed in this cohort of 20 patients were otitis media (121 episodes) and pneumonia (61 episodes), followed by conjunctivitis, arthritis, sepsis, sinusitis, and meningitis (Figure 2). The mean IgG level before IVIG therapy was 0.58 g/l (range, 0.01–3.17 g/l).

Pathogenic mutations in the *BTK* gene were identified in 17 of our cohort of 20 unrelated symptomatic patients tested by sequencing the 18 coding exons and exon–intron junctions. The mutations included missense and nonsense mutations, insertions, deletions, and splicing defects and were located throughout the *BTK* gene (Table 1; Figure 1). Additional samples were provided from 15 mothers of the 17 unrelated patients with *BTK* mutations (Table 2). Thirteen of the mothers had the same mutation as their sons and are considered XLA carriers. Of three girls, each having a full brother with a *BTK* mutation, one was found to be a carrier. The daughter of one of the two mothers with normal BTK sequence was found to have the same *BTK* mutation as her brother (p01), suggesting gonadal mosaicism.

**Table 2:** Carriers found among mothers and sisters of patients with XLA diagnosis.

Patient	Relative tested
p01^a^	Mother not carrier^b^
	Sister carrier
p02	Mother carrier
p03	Mother not carrier^b^
p04	Mother carrier
p05	Mother carrier
p06	Mother carrier
p07	Mother carrier
p08	Mother carrier
p11	Mother carrierSister not carrier^b^
p12	Mother carrierSister not carrier^b^
p13	Mother carrier
p14	Mother carrier
p15	Mother carrier
p16	Mother carrier
p17	Mother carrier

*^a^The presence of a BTK mutation in the brother (p01) and sister while absent in the mother suggests gonadal mosaicism of the mother*.

*^b^Target sequencing directed to the affected exon of the BTK gene did not reveal the mutation of the proband*.

*BTK, Bruton tyrosine kinase*.

## Discussion

The diagnosis of agammaglobulinemia can be suspected based on the clinical and laboratory characteristics. While the majority of patients with agammaglobulinemia are male, suggesting X-linked inheritance and mutations in *BTK*, there are a handful of genes that, if mutated, result in autosomal recessive agammaglobulinemia, including *IGHM* (μ heavy chain deficiency), *IGLL1* (λ5 deficiency), *CD79A* (Igα deficiency), *CD79B* (Igβ deficiency), *BLNK, PIK3R1* (1), *IKAROS* (16), and one gene (*TCF3*, E47 deficiency) causing an autosomal-dominant agammaglobulinemia (17). Because of this heterogeneity, molecular analysis is imperative for optimal therapy, prognostic considerations, and genetic counseling of patients with agammaglobulinemia. While sequence analysis, either by Sanger sequencing of individual genes or by next-generation sequencing is broadly available in the developed world, most developing countries do not have access to these advanced techniques.

This pilot study demonstrates the feasibility of collecting blood on Guthrie cards, shipping the cards at minimal cost from one continent to another, and identifying causative mutations in *BTK* by Sanger sequencing in 17 of 20 (85%) unrelated male patients with clinical and laboratory findings compatible with XLA. We have used this technique to collect and ship DBS from a number of South American countries to identify disease-causing mutations in >25 PID-associated genes including *WAS, CD40L, PIK3CD, FOXP3, CTLA4, STAT1, STAT3, RAG1*, and *RAG2*. Because of the limited quantity of patient material, we had to slightly modify the PCR amplification technique that we routinely use for gDNA extraction from peripheral blood or saliva.

The type and distribution of the *BTK* mutations were similar to those reported previously. Since only the coding region and the intron/exon junctions of *BTK* were sequenced, mutations in the promoter or Poly A regions, or deep intronic variants, would have been missed. Identifying those mutations will require analysis of mRNA or protein expression, we hope to develop in the future. The clinical characteristics and laboratory findings of the 17 Vietnamese XLA patients with BTK mutations were similar to those observed in Western countries, with episodes of pneumonia reported by 80% of the Vietnamese patients, 62% of US patients (7), 100% of patients from the Netherlands (18), and 77% of patients from China (8).

The mean age at diagnosis, based on clinical and immunological criteria, was similar to Western countries: 4.6 years in Vietnamese patients, compared with 5.37 in the United States (7) and 6.5 years in patients from the Netherlands (18), but slightly lower than in those from China (7.09 years) (8).

In conclusion, DBS are easy to collect, can be stored and shipped at room temperature, and transported inexpensively across continents. DBS are an excellent source of high-quality gDNA in sufficient quantity for Sanger sequencing, making this collection technique an affordable option for developing countries to interact and collaborate with sophisticated laboratories anywhere in the world.

## Ethics Statement

Institutional review boards (IRBs) at Seattle Children’s Hospital and Vietnam National Children’s Hospital.

## Author Contributions

HO and TT conceived and designed the study; GS developed the technique and sequenced BTK from DBS; AN, HTML, HT, LN, and HTL identified the patients and collected DBS; RK established contact with the Hanoi group and facilitated the shipment of samples.

## Conflict of Interest Statement

The authors declare that the research was conducted in the absence of any commercial or financial relationships that could be construed as a potential conflict of interest.
